# Role of Montelukast in Improving Quality of Life in Patients with Persistent Asthma

**DOI:** 10.7759/cureus.5046

**Published:** 2019-06-30

**Authors:** Ayesha Ikram, Vinod Kumar, Muhammad Taimur, Mahrukh A Khan, Sundus Fareed, Habiba D Barry

**Affiliations:** 1 Internal Medicine, King Edward Medical University/Mayo Hospital, Lahore, PAK; 2 Hospital Medicine, Cleveland Clinic Abu Dhabi, Abu Dhabi, ARE; 3 Internal Medicine, Dow University of Health Sciences, Karachi, PAK; 4 Internal Medicine, Civil Hospital Karachi, Karachi, PAK

**Keywords:** montelukast, quality of life, asthma control test, maintenance therapy, persistent asthma, leukotriene receptor antagonist, asthma treatment, controller therapy

## Abstract

Introduction

Maintenance therapy of asthma has a crucial role in keeping the disease dormant and preventing frequent acute exacerbations. Asthma control may be achieved by inhaled corticosteroids (ICS) and/or long-acting beta-agonists (LABA). Leukotriene receptor antagonist - montelukast - may be added as an add-on to ICS/LABA or may also be given in monotherapy. The aim of this study was to evaluate the role of montelukast monotherapy as asthma control and its impact on the quality of life of these patients.

Methods

In this prospective, open-label, interventional study, montelukast 10 mg once daily was given to patients with mild to moderate persistent asthma for four weeks. Quality of life (QOL) was assessed on the Asthma Quality of Life Questionnaire - Standard (AQLQ-S) questionnaire. Asthma control was assessed on the Asthma Control Test (ACT). Data was entered and analyzed using SPSS version 23.0.

Results

On AQLQ-S, overall QOL improved with one month of montelukast therapy significantly. On sub-scales, except for emotional function, all other three sub-scales including symptoms, activity limitation, and environmental function improved significantly. Asthma control score also significantly improved with one month of montelukast therapy.

Conclusion

Montelukast has an effective role in asthma control and improvement of QOL in patients with mild to moderate persistent asthma.

## Introduction

The chronic airway obstruction with hyper-responsiveness, which is due to immunologically mediated airway remodeling, is known as asthma. Its etiology is multifactorial with complex genetic actions and environmental triggers playing an interchangeable role [1]. Asthma is among the most common chronic non-communicable diseases. It is estimated that in 2018, 339 million people are affected by asthma globally. Uncontrolled and inadequately managed asthma substantially increases the burden of reduced quality of life (QOL) and also premature death. Asthma has been ranked 16th among the leading causes of years lived with disability and 28th among the leading causes of burden of disease, as measured by disability-adjusted life years (DALYs) [2].

The cardinal respiratory symptoms of asthma - cough, chest tightness, wheezing, and shortness of breath - are caused by airway inflammation. The pathophysiology of asthma, which contributes to its chronicity, is complex. Inflammation of the airways in asthma involves many types of proinflammatory cells and mediators. These include eosinophils, mast cells, neutrophils, macrophages, T lymphocytes, dendritic cells, and airway epithelial cells. T helper 1 cells and T helper 2 cells (Th2) are also activated in this cascade. Th2 generate cytokines including interleukin [IL]-4, IL-5, and IL-13, which induce the production of immunoglobulin E (IgE) and eosinophils and promote airway hyperresponsiveness. Mucosal mast cells release the bronchoconstricting mediators including histamine, prostaglandins, and cysteinyl leukotriene (CysLTs) that are potent bronchoconstrictors [3]. The activity of CysLTs also causes smooth muscle contraction, enhanced leakage from the vasculature leading to respiratory edema, decreased mucociliary clearance, increased mucus production, and also attracts leukocytes to augment the inflammatory process. Airway remodeling in chronic asthma is mediated by CysLTs as they stimulate the airway smooth muscle and epithelial proliferation and increases the deposition of collagen [4-5].

The Global Initiative for Asthma (GINA) guidelines (2018) recommend leukotriene receptor antagonists (LTRAs) as monotherapy in mild-persistent asthma and as an add-on or alternative to increasing dose of inhaled corticosteroids (ICS) or adding a long-acting β2-agonist (LABA) [6]. LRTAs have shown efficacy in long-term control of asthma exacerbations; however, the gold-standard for asthma control has been ICS [7]. Montelukast is a CysLRTA, which has a key role in inhibiting bronchoconstriction and relieving the inflammatory process [8]. Montelukast has been studied as an add-on therapy to ICS for long-term control of mild to moderate persistent asthma [9-11]. The aim of this study was to evaluate the role of montelukast monotherapy in control of mild to moderate persistent asthma in adults.

## Materials and methods

This prospective, open-label, interventional study was conducted for three months (January to March 2019) in the outpatient department (OPD) of the pulmonology department of a tertiary-care hospital in Lahore, Pakistan. The study was approved by the institutional ethical committee and informed consent of participation was taken from all patients. All patients, of both genders and of age 15 years and above, diagnosed with asthma for at least one year, attending the OPD for routine follow-up visits, who were naïve to montelukast were consecutively invited to participate. All patients were prescribed montelukast (10 mg once daily) by the treating pulmonologist. No ICS or LABA was added.

All participants completed the study questionnaire twice - once at the start of the study (day 0) and second after one month (day 30). Patient demographics included in the study were age, gender, duration of asthma, and frequency of asthma exacerbations. All participants completed two standard instruments to evaluate the extent of asthma control and improvement in the quality of life. Asthma control test (ACT) was used to assess the efficacy of treatment [12]. It is a five-item instrument that evaluates asthma control. It was completed by the participants once at the start of the study and then after one month. On ACT, mean score <20 signifies uncontrolled asthma, 20-24 signifies adequate control, and a score of 25 signifies excellent control. Asthma Quality of Life Questionnaire - Standard (AQLQ-S) was used in this study to evaluate the QOL in asthmatic patients [13]. It is a 32-item instrument with four sub-domains - symptoms, activity, emotions, and environmental control. Each sub-domain and the overall score is marked on a seven-point Likert scale. A higher score indicated higher QOL and better ambulatory functioning.

Participants with less than one year of asthma diagnosis, presenting with acute exacerbation of asthma, and those who had previously taken montelukast for asthma control were excluded from the study. Participants who reported any adverse event of montelukast during the study period were also excluded (and montelukast was stopped). Participants who filled incomplete questionnaires and/or could not be tracked after one month of treatment were also excluded from the analysis.

Data were processed through and analyzed using SPSS for Windows version 23.0 (IBM, USA). Mean and standard deviation (SD) were calculated for continuous variables including age and AQLQ-S and ACT score. Frequency and percentage were calculated for categorical variables including gender, age, duration of asthma, and frequency of exacerbations. A comparison was done for scores of AQLQ-S and ACT on days 0 and 30 using dependent *T*-test. *P-*values ≤ 0.05 were taken as statistically significant.

## Results

At the start of the study, 112 participants were included. During the study period, two (1.8%) participants withdrew montelukast due to adverse events, three (2.7%) participants withdrew from the study (no reason stated), and 14 (12.5%) participants were lost to the second follow-up visit. The study was completed by 93 (83.0%) participants. Their mean age was 23.6 ± 8.4 years, 59 (63.4%) of them were men, and 34 (36.5%) were women. Their demographic and disease characteristics are shown in Table 1.

**Table 1:** Patient age, gender, duration of asthma, and frequency of asthma exacerbations (N = 93) SD, standard deviation

Patient Characteristics	Frequency (%)
Gender	
Male	59 (63.4%)
Female	34 (36.5%)
Age in years	
Mean ± SD	23.6 ± 8.4
15-25 years	48 (51.6%)
26-35 years	29 (31.1%)
≥ 36 years	16 (17.2%)
Duration of asthma	
< 1 year	8 (8.6%)
1-3 years	23 (24.7%)
3-6 years	37 (39.7%)
≥ 7 years	25 (26.8%)
Frequency of asthma exacerbations	
≥ 1 time / month	33 (35.5%)
≥ 1 time / 3 months	28 (30.1%)
≥ 1 time / 6 months	21 (22.5%)
≥ 1 time / year	11 (11.8%)

The mean scores of both scales - AQLQ-S and ACT - were compared for day 0 and day 30. On AQLQ-S, overall QOL improved with one month of montelukast therapy improved significantly. On sub-scales, except for emotional function, all other three sub-scales including symptoms, activity limitation, and environmental function improved significantly. Asthma control score also significantly improved with one month of montelukast therapy, as shown in Table 2.

**Table 2:** Mean score of AQLQ-S and ACT at the start of the study and after one month of therapy (N = 93) SD, standard deviation; AQLQ-S, asthma quality of life questionnaire – standard; ACT, asthma control test * Dependent T test applied. *P* ≤0.05 taken as significant

Study Instruments	At Day 0 (Mean ± SD)	At Day 30 (Mean ± SD)	P value*
AQLQ-S Score			
Overall	4.04 ± 0.66	5.24 ± 0.93	<0.0001
Symptoms	3.84 ± 0.72	4.12 ± 0.37	0.001
Activity limitation	4.46 ± 1.76	5.09 ± 0.94	0.002
Emotional Function	4.43 ± 1.95	4.59 ± 1.14	0.49
Environmental Function	4.67 ± 1.08	5.33 ± 1.48	0.0006
ACT Score			
	17.59 ± 1.79	21.52 ± 2.17	<0.0001

## Discussion

This study has highlighted the role of montelukast therapy in control of asthma and in improving the overall quality of life in these patients. This study has produced substantial results and contributes efficiently to the existing literature entailing the role of montelukast in chronic asthma. However, this study has its limitations too. It was an open-label study and comparison was not done with placebo. Furthermore, all patients were montelukast naive. For more long-term impacts, longitudinal studies are recommended with double-blind, randomized study designs.

The landmark trial to study the role of montelukast in asthma was MOntelukast in Chronic Asthma (MONICA) trial. In this trial, montelukast 10 mg daily was added to ICS and/or LABA for six months. Improved scores were seen on both ACT and mini-AQLQ [9]. In a meta-analysis of 50 trials, the risk of asthma exacerbation was reduced to 0.60 in six trials with LTRA monotherapy. In four trials with LTRAs add-on therapy to ICS, the risk ratio for exacerbation was 0.80 [6]. In Bozek et al., after two years of treatment, the median number of asthma exacerbation incidents decreased from 1.6 per year per patient to 1.2 per year per patient when montelukast was added to ICS and LABA [10]. Biernacki et al. also reported significant improvement in the AQL questionnaire when montelukast was added to ICS [11].

On the other hand, there are studies that have failed to produce any substantial improvement in asthma control with montelukast therapy. One of such studies was conducted in elderly patients, and ACT scores and biochemical assay were recorded at weeks four and eight of montelukast therapy. The results were not statistically significant [14]. In another randomized placebo-controlled trial, there was no improvement of symptoms of acute asthma with the addition of montelukast [15].

In a Korean study, adding montelukast to low-dose ICS showed better results in mild to moderate persistent asthma as compared to increasing the dose of ICS only. ACT scores and forced expiratory volume in one minute improved and frequency of acute asthma exacerbations reduced [16]. In a local trial, montelukast monotherapy was compared with ICS monotherapy in chronic asthma. In the first and the third week, peak expiratory flow rate was better in the montelukast group (*p* <0.05), from fourth till eight weeks; it was similar for both groups [17]. In another local trial, montelukast monotherapy was compared with placebo. Montelukast group did not show significant improvement on the ACT but AQLQ-S, overall and on sub-domain “environmental stimuli” improved significantly [18]. In a recent Chinese study, montelukast played a substantial role in regulating Th1/Th2 cell balance and increased the expression of CD4+ CD25+ regulatory T cells. This aided in the alleviation of airway inflammation and helped in improving the clinical symptoms as well as the lung function of these patients [19].

Although acting through different mechanisms, montelukast has been shown to be equivalent to tiotropium as an add-on to ICS + LABA in asthma. Both the drugs improved airway obstruction and pulmonary function [20]. In a meta-analysis of 20 trials, montelukast as an add-on to ICS/LABA in adults with chronic asthma significantly reduced the number of exacerbations (odds ratio = 0.60). In adults with acute asthma, montelukast statistically improved peak expiratory flow and reduced the need for systemic corticosteroids [21].

The role of montelukast in mild to moderate persistent asthma as well as in acute asthma has been supported by substantial literature. It helps in alleviating the airway inflammation, improving clinical signs, reducing the frequency of exacerbations, and improving the overall quality of life of these patients. Montelukast should be considered by physicians as an add-on to ICS/LABA and in patients where ICS and/or LABA fail to produce adequate asthma control. Longitudinal, randomized, double-blind trials must be initiated to evaluate the effect of montelukast in comparison to placebo as well as in comparison to ICS and/or LABA in persistent asthma. Montelukast has the benefit of once daily dose and the treatment regime is cheaper as compared to ICS and LABA, hence will have better patient compliance. More evidence should be focused on whether montelukast can be promoted to “ICS sparing agent” or not. There is still scarce data on the effect of montelukast on asthma-related mortality.

## Conclusions

Montelukast has an effective role in asthma control as well as in improving the quality of life in patients with mild to moderate persistent asthma. Montelukast can be a promising alternate to ICS/LABA and/or as an add-on to ICS/LABA in chronic asthmatic patients with uncontrolled symptoms. More focused randomized trials must be initiated to evaluate the role of montelukast as an “ICS sparing agent” in mild to moderate persistent asthma.
